# Amebiasis of the Gallbladder: A Rare Presentation

**DOI:** 10.7759/cureus.92837

**Published:** 2025-09-21

**Authors:** Eshaan Chandra, Mohit K Badgurjar, Geeta W Mukhiya, Suman Parihar, Neha Pandya

**Affiliations:** 1 General Surgery, Geetanjali Medical College and Hospital, Udaipur, IND; 2 Pathology, Geetanjali Medical College and Hospital, Udaipur, IND

**Keywords:** amebiasis, cholecystectomy, entamoeba histolytica, gallbladder, histopathology, metronidazole

## Abstract

Amebiasis is caused by *Entamoeba histolytica*, commonly found in the liver and colon. Its presentation is rare in the gallbladder. A 25-year-old female presented with epigastric pain radiating towards the right hypochondrium. The patient was diagnosed with calculous cholecystitis. Later on, performing a laparoscopic cholecystectomy, histopathology resulted in acute-on-chronic necrotizing cholecystitis with *E. histolytica* trophozoites. The treatment included metronidazole 500 mg thrice daily for 14 days, resulting in full healthy recovery. This case report highlights the importance of considering a histopathological examination when evaluating rare gallbladder pathologies and treating them with accurate treatment.

## Introduction

*Entamoeba histolytica* is a parasite that remains a global health burden, mainly in developing countries, despite advancements [1]. It is generally found in areas with poor sanitation, poverty, and poor dietary habits. Amoebae multiply and block small intrahepatic portal radicles, followed by focal infarction of hepatocytes. It is commonly found in the colon and liver [1]. Its location in the gallbladder is extremely rare [2]. Treatment of amebiasis is generally based on severity through amebicides. Metronidazole is widely used in the treatment of amebiasis due to its high efficacy and low cost [3]. Its dose must be 750-800 mg three times a day orally for 10 days in adults and 50 mg/kg/day in children. Only two other cases have been found in the literature. In both reported cases, it was diagnosed in histopathology after cholecystectomy [2,4]. The aim of this case report is to highlight the rare possibilities of gallbladder pathologies and to emphasize the importance of histopathological examination after laparoscopic/open cholecystectomy.

## Case presentation

**Figure 1:**
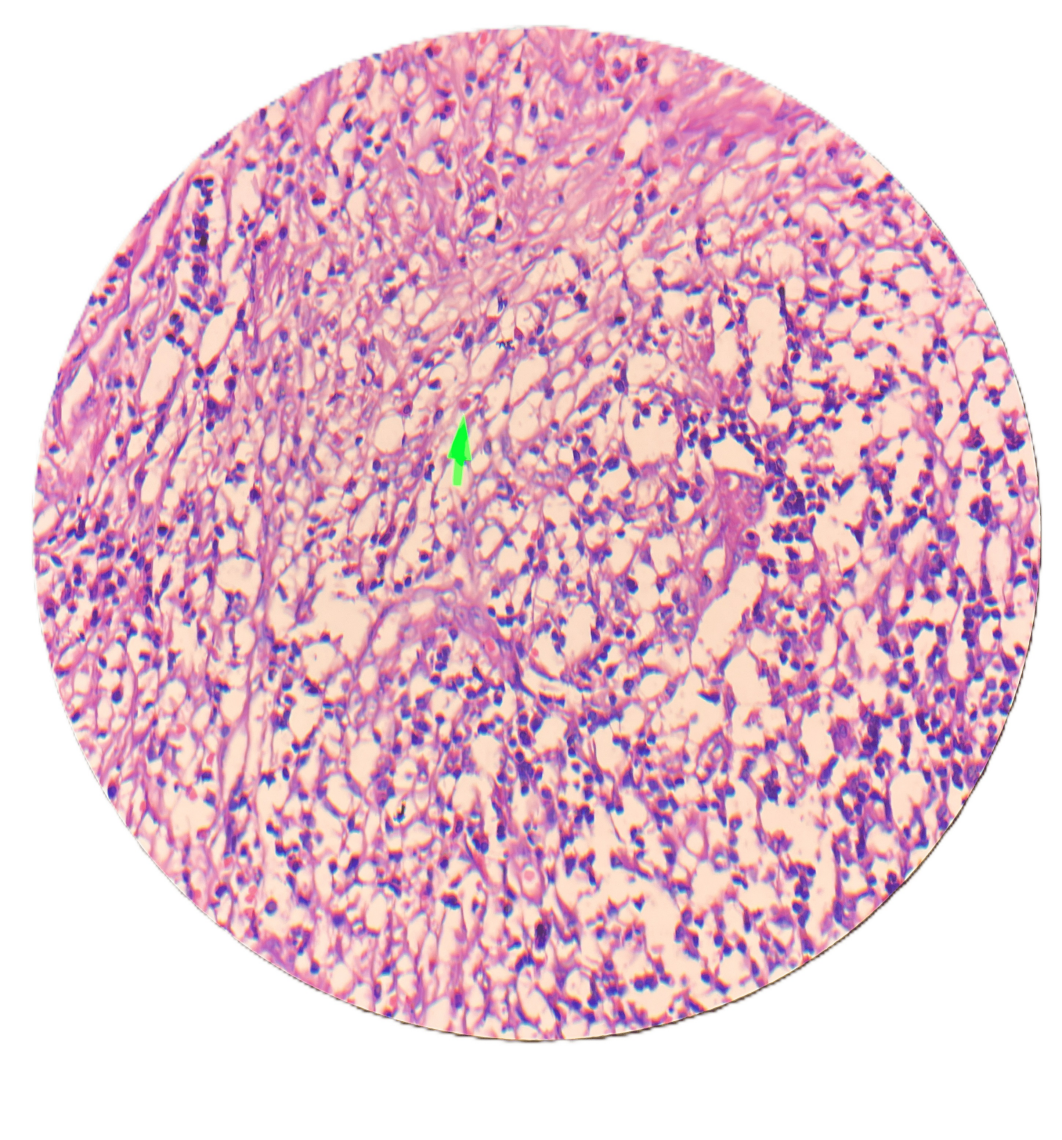
Scanner view (4× magnification, H&E stain) showing numerous eosinophils and neutrophils (green arrow). HE: hematoxylin and eosin

**Figure 2:**
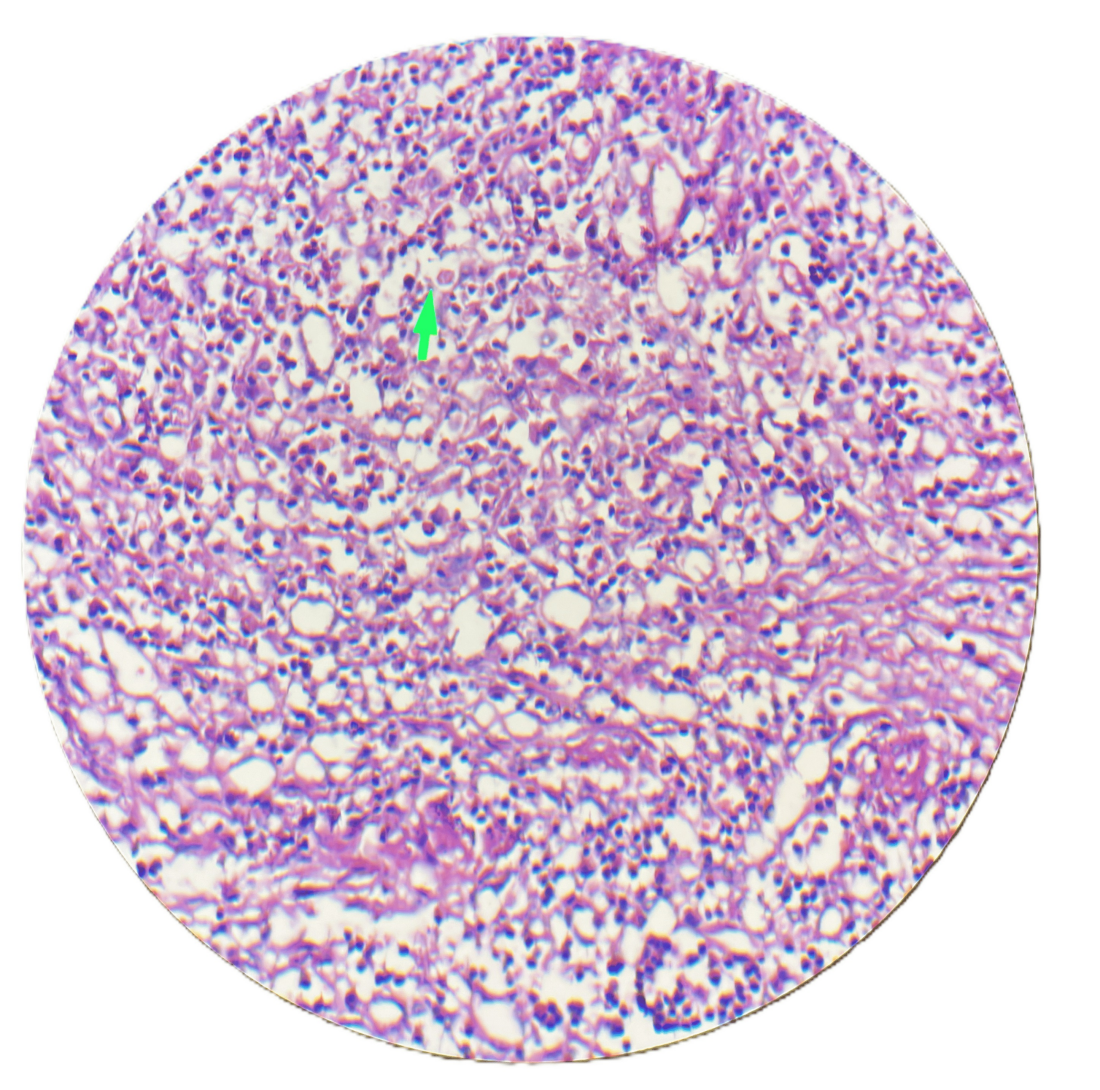
Scanner view (4× magnification, H&E stain) showing trophozoites of Entamoeba histolytica in the lamina propria of the gallbladder, along with numerous neutrophils and eosinophils (green arrow). HE: hematoxylin and eosin

**Figure 3:**
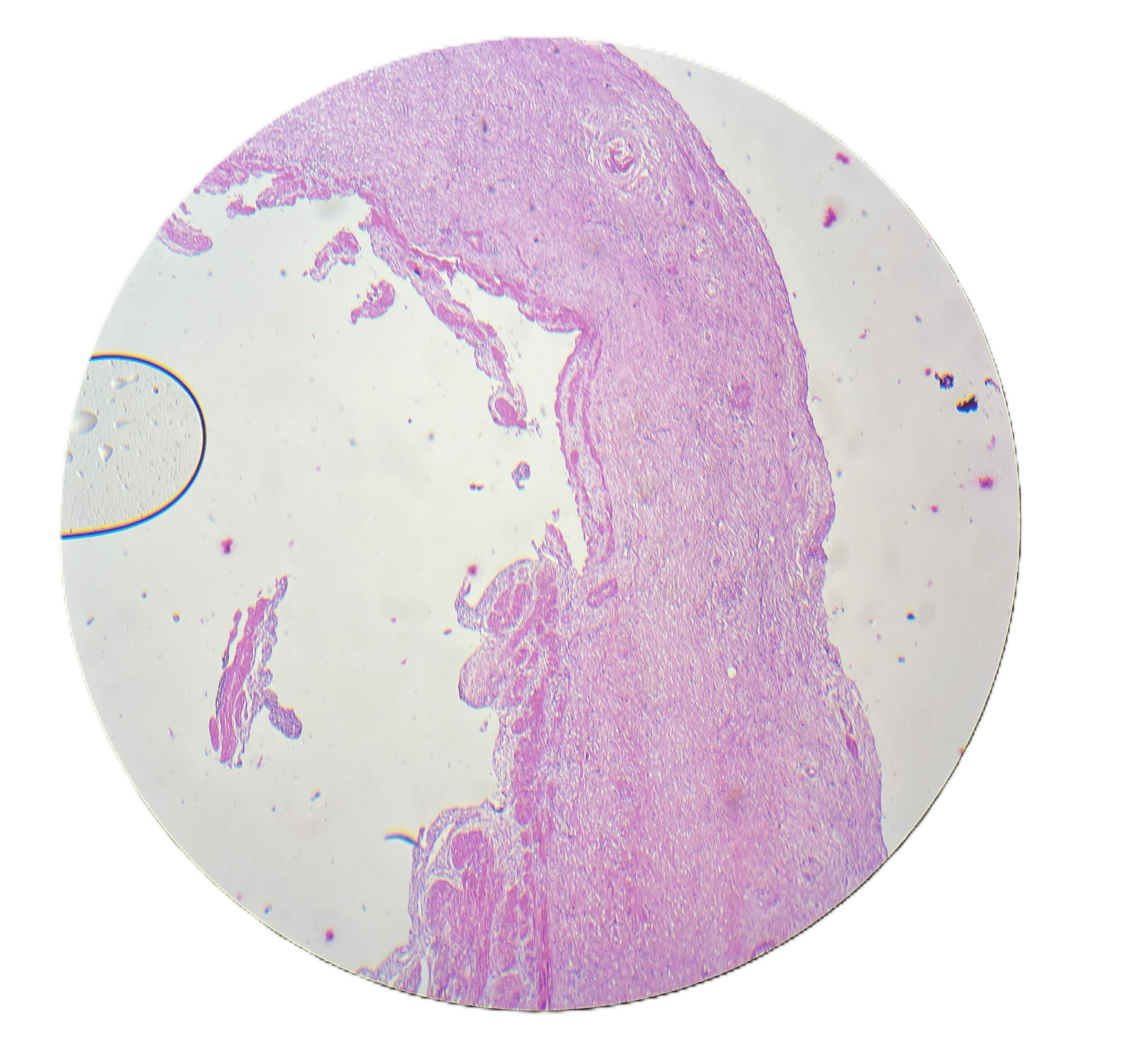
Scanner view (4× magnification, H&E stain) showing necrosis of the gallbladder mucosa. HE: hematoxylin and eosin

**Figure 4:**
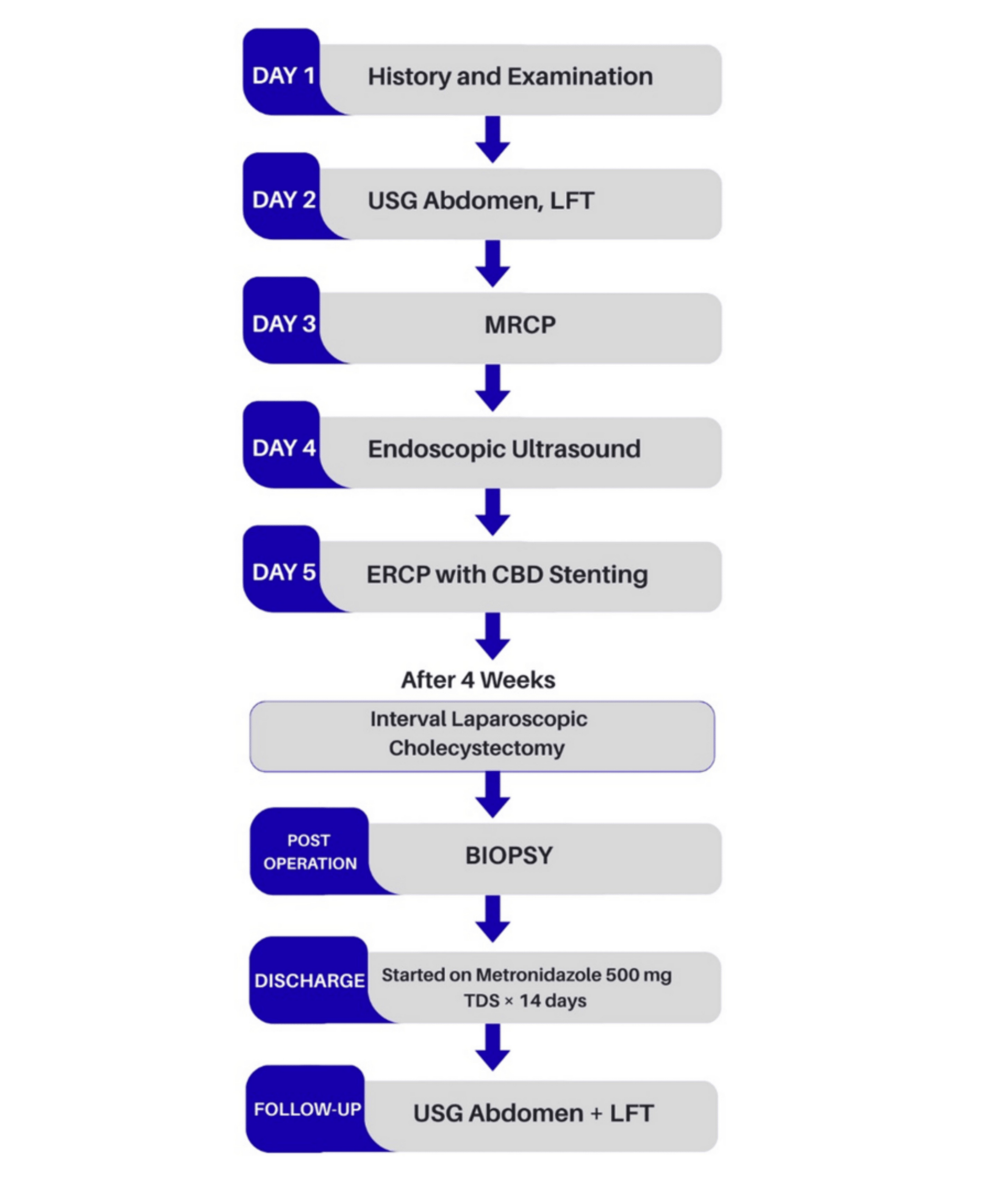
Flowchart of the patient's diagnostic process. USG: ultrasonography; LFT: liver function test; MRCP: magnetic resonance cholangiopancreatography; ERCP: endoscopic retrograde cholangiopancreatography; CBD: common bile duct; TDS: Ter in die sumendum (three times a day)

A 25-year-old female patient presented in November 2023 with complaints of pain in the epigastric region radiating to the right hypochondrium for 15 days. There was no history of dysentery, vomiting, fever, or weight loss. On examination, the patient was in good general condition without any tenderness or palpable mass over the abdomen. An ultrasound of the whole abdomen was suggestive of cholelithiasis. Liver function tests (LFTs) were deranged with an increase in alkaline phosphatase. Further, magnetic resonance cholangiopancreatography (MRCP) was done to rule out choledocholithiasis. MRCP was suggestive of cholelithiasis with cholecystitis. To further investigate the cause of persistently increased alkaline phosphatase, endoscopic ultrasound was performed, which suggested common bile duct (CBD) sludge or CBD stone, and endoscopic retrograde cholangiopancreatography (ERCP) was done with CBD stenting. An interval laparoscopic cholecystectomy was performed after four weeks of ERCP. Intraoperatively, adhesions were found between the gallbladder and duodenum, with a thickened gallbladder wall. The gallbladder was excised and sent for biopsy. The patient had an uneventful post-operative period of two days and was discharged comfortably. Biopsy results were suggestive of acute-on-chronic necrotizing cholecystitis with cholelithiasis, showing numerous eosinophils and neutrophils, with inflammation extending to involve the muscularis mucosa (Figure 1), trophozoites of *E. histolytica* along with numerous neutrophils and eosinophils (Figure 2), and necrosis of the mucosa of the gallbladder wall (Figure 3). In view of amebiasis, the patient was advised to take metronidazole 500 mg thrice a day for 14 days. Ultrasonography (USG) of the whole abdomen and LFT were repeated after treatment and were found within normal limits. The patient was called after four weeks of surgery for stent removal. The patient remained stable and had no complaints at the two-month follow-up. A flowchart or timeline of the patient’s diagnostic process is presented in Figure 4.

## Discussion

Amebiasis is the fourth leading cause of mortality from parasitic diseases worldwide, caused by the human parasite *E. histolytica* [1]. The first and most common mode of transmission is swallowing cysts coming from dirty or infected water or food. Venereal transmission also occurs through the fecal-oral route [5]. Amebiasis may present as an asymptomatic carrier state or mild to severe symptoms, depending upon the location [6]. The symptoms of amebiasis are classified into two types: intestinal symptoms, which directly impact the digestive system, and extra-intestinal symptoms, which indirectly impact the remaining parts of the body, excluding the intestines. The most common site in the intestine is the colon, presenting with fulminant colitis, bloody-tinged diarrhoea, fever, and weight loss. Liver abscess is the most common extra-intestinal manifestation of the disease [7]. An amebic liver abscess can spread to different areas of the body, including the thorax, resulting in empyema, and can also spread to the pericardium, which can be extremely dangerous [8].

Other rare sites can be the gallbladder, brain, and skin. There are reports in which amebiasis presents as a mass, referred to as an ameboma, which is a pseudotumor. It can occur in the colon, mimicking colonic cancer, and in the liver, mimicking liver cancer [2]. Gallbladder amebiasis is an extremely rare finding, as only two other cases have been reported earlier [2,4]. In the reported case by Ouadi et al. [2], gallbladder amebiasis presented as an amoeboma mimicking cholangiocarcinoma with biliary colic and suspicion of malignancy on imaging. In another report by Ben Abid et al. [4], three cases of pseudo-tumoral digestive amebiasis were described, one of which involved the gallbladder and was initially suspected to have a neoplastic etiology. Whereas in our case, the patient mainly had symptoms of gallbladder stones, without any signs or symptoms of amebiasis or malignancy. Hence, in the other two reported cases and even in our case, amebiasis was diagnosed after the histopathology report. If not identified histopathologically, there is a theoretical chance that remnant infestation of amebiasis may spread to the rest of the biliary tree. This may cause the production of sludge or even ameboma in the biliary tract, leading to biliary obstruction [9]. Therefore, proper histopathological examination is always important to diagnose such rare pathology so that we can provide the further required treatment, which was metronidazole in this case [10]. All the reported cases, including ours, were managed by cholecystectomy followed by metronidazole therapy.

## Conclusions

Amebiasis of the gallbladder is a rare and exceptional entity. After cholecystectomy, the gallbladder should be sent for biopsy to rule out such rare diseases. A thorough histopathological study is very important as it helps the surgeon to reach a final diagnosis and plan the next line of management. In this case, metronidazole is used to eliminate the parasite and prevent the spread of the disease.
